# Supplementary material for: Neurosensory Rehabilitation and Olfactory Network Recovery in Covid-19-related Olfactory Dysfunction
Source: Brain Sci. 2021 May 23;11(6):686. doi: 10.3390/brainsci11060686 (PMC8224593; doi:10.3390/brainsci11060686)
Supplement: Supplementary file 1 [file brainsci-11-00686-s001.zip › brainsci-1221617-supplementary.pdf]

brain sciences

MDPI

Supplementary Materials (Appendix)

Supplementary Notes

Supplementary Note S1: Olfactory evaluations and SARS-CoV-2 virologic assessments.

Supplementary Note S2: Neuroradiological magnetic resonance imaging (MRI) data acquisition.

Supplementary Note S3: Smell training via the electronic portable aromatic rehabilitation (EPAR) diffusers.

Supplementary Tables

Supplementary Table S1: Demographic and clinical characteristics of COVID-19 patients and controls.

Supplementary Table S2: Sino-nasal outcome test (SNOT-22) of patient 1, before olfactory treatment.

Supplementary Table S3: Sino-nasal outcome test (SNOT-22) of patient 1, after 4-weeks of olfactory treatment.

Supplementary Table S4: Sino-nasal outcome test (SNOT-22) of patient 2.

Supplementary Table S5: Functional connectivity (FC) of the left and right caudate and the olfactory cortical network (OCN) regions.

Supplementary Table S6: Volume of the olfactory bulb and tract.

Supplementary Table S7: Cerebral blood flow (CBF) in the olfactory cortical network (OCN) regions.

Supplementary Table S8: Butanol threshold test (BTT).

Supplementary Figures

Supplementary Figures S1 | Magnetic resonance (MR) spectroscopy of the gyrus rectus and superior frontal cortex. (A) The position of the single voxel Point Resolved Spectroscopy (PRESS) was placed at the gyrus rectus and superior frontal cortex. (B) Healthy control. (C) Patient 2. Cho, choline; Cr, creatine; mI, myo-inositol; NAA, N-acetylaspartate.

Supplementary Figures S2 | Electronic portable aromatic rehabilitation (EPAR) diffuser. (A) Correct usage, stream of aerosolised essential oil directed vertically upwards, away from the user’s face. (B) Incorrect usage, stream of

aerosolised essential oil directed at the user's face, which may cause transient irritation to the skin and mucosal surfaces.

Supplementary Item

Supplementary Item S1: Olfactory function questionnaire.

Supplementary References

Supplementary Note S1: Olfactory evaluations and SARS-CoV-2 virologic assessments.

Patients and controls

All participants completed a detailed olfactory questionnaire at study recruitment (Supplementary Item S1).[1 2]

Olfactory evaluations

Subjective olfactory performance was assessed using Sino-Nasal Outcome Test (SNOT-22; Supplementary Tables 1–3).[3] Quantitative olfactory function was measured using butanol threshold test (BTT; prepared in-house; Supplementary table 8) and smell identification test (SIT; Sensonics International, New Jersey, USA).[4 5] Nasoendoscopic examinations were performed for COVID-19 patients, as described previously.[1]

SARS-CoV-2 reverse transcription polymerase chain reaction (RT-PCR) tests

COVID-19 patients were diagnosed by RT-PCR of pooled nasopharyngeal and throat swab specimens targeting the SARS-CoV-2 envelope gene (TIB Molbiol, Berlin, Germany). Control subjects were tested negative for SARS-CoV-2 by commercial PCR assays (Xpert® Xpress SARS-CoV-2, Sunnyvale, California, United States; or RT-PCR COVID-19 Test, Prenetics, Hong Kong, China) at the time of MRI evaluation.

SARS-CoV-2 serological assessments

All study participants received SARS-CoV2 serological assessments by enzyme immunoassay (EIA) tests for SARS-CoV-2 nucleoprotein (NP) and spike protein receptor binding domain (RBD); furthermore, microneutralization assays (MN) were performed, as previously described (Table 1).[6 7]

Supplementary Note S2: Neuroradiological magnetic resonance imaging (MRI) data acquisition.

All subjects underwent MRI brain scans using a 1.5T MR scanner (SIGNA; GE Healthcare, Chicago, Illinois, United States) with a standard 16-channel head coil. Structural MRI brain images were acquired with fast and high-resolution three-dimensional (3D) sequences (BRAVO_3D sagittal, TI=450ms, Flip angle=13°, voxel size=1×1×1.2mm^{3}, FOV=256×256mm^{2}). The parameters of the 3D arterial spin labeling (ASL) were TR=4885ms, TE=10.5ms, labeling-duration=1800ms, post-labeling delay=2025ms, 40 slices with spatial resolution of 1.9×1.9×4mm^{3}.

Volumetric analyses of the olfactory bulbs and tracts included a sagittal 3D T2 FLAIR (TR=6000ms, TI=1858ms, FOV=24cm) with 1.4mm interleaved scans. After reformatting, the coronal plane was obtained with 1.5mm spacing. The 3D volumetric processing of the bilateral olfactory bulbs and tracts were performed on GE workstation (AW server 3.2; GE Healthcare, Chicago, Illinois, United States). The olfactory bulbs were located at the anterior cribriform plate, while the olfactory tracts extended posteriorly to enter the brain below the rostrum of the corpus callosum.[8]

MR spectroscopy was performed using the single voxel Point Resolved Spectroscopy (PRESS), TE=136ms, TR=1500ms, voxel size=2×2×2cm^{3}.[9] The voxel was placed at gyrus rectus (GR) and superior frontal cortex.

Resting state functional magnetic resonance imaging (rs-fMRI) of the brains were collected using a gradient-echo echo-planar sequence (parameters: TR=2000ms, TE=30ms, flip angle=90°, voxel size=3×3×4mm^{3}) sensitive to blood-oxygen-level-dependent (BOLD) contrast. During the rs-fMRI scanning, the participants were instructed to open their eyes to look at the cross presented within the MR scanner and not to think of anything.

The pre-processing of the rs-fMRI data were performed using the DPABI toolbox (http://rfmri.org/dpabi) based on the SPM12 software (https://www.fil.ion.ucl.ac.uk/spm/software/spm12/). The first ten images were discarded to account for the spin saturation effects and acclimatization of scanning noise by the subjects. The differences in image acquisition time of the remaining rs-fMRI images were corrected between slices. Head motion corrections were performed to adjust

the images in the same position. Images were excluded from further analyses if the head movements were greater than 3mm in any dimensional planes of x, y, or z; or over 3° of deviation. Nuisance signals, including Friston-24 head motion parameters, mean white matter (WM), and cerebrospinal fluid (CSF) time series within brain masks were regressed out from the time courses in each voxel. Then the images were spatially normalized to the standard Montreal Neurological Institute (MNI) space and resampled to $3 \times 3 \times 3 mm^3$ using the transformation parameters that were estimated through DARTEL segmentation.[10] After normalization, the data were band-pass filtered ($0.01 < f < 0.1$Hz) to reduce the high-frequency respiratory and low-frequency cardiac noise drifts.

Hypothesis-driven region of interest (ROI) approach was applied in the evaluation of the rs-fMRI images. The centers of the seed regions were located at the MNI coordinates for the left (-14, 14, 2) and right (14, 12, 9) caudate nuclei, respectively.[11 12] We defined the seed regions for functional connectivity (FC) analyses with a sphere of 10 mm radius. We then calculated the correlations between the ROI series and the whole brain for each individual subject in a voxel-wise manner. To normalize the distribution of correlation coefficient (Pearson correlation, *r*), the values were transferred to standard z scores based on Fisher transformation. The connectivity maps of patients and healthy controls of similar age groups were calculated.

Based on the Automated Anatomical Labeling (AAL) template, the pre-processed rs-fMRI data were segmented into 90 regions.[11] Twenty-eight out of the 90 regions were associated with the functional olfactory cortical networks (OCN). The primary OCN areas included the bilateral piriform gyrus and amygdala; while the secondary OCN areas included the superior orbitofrontal cortex (OFC), inferior OFC, medial OFC, GR, insula, anterior cingulate cortex, hippocampus, parahippocampus, caudate nucleus, putamen, as well as the superior and middle temporal poles.[12-14] We created a mask which included both primary and secondary OCN processing areas (Fig. 1). The results provided spatial maps of the

olfactory network FC within the created mask. The threshold was set at 0.2 and maximum z value was standardized to 1.

Based on the ROIs of olfactory regions reported the connectivity between seed regions (left and right caudate) and other ROIs were obtained by extracting the average time series from each ROI and calculating the correlation.[15-17] The average FC values between seeds and other regions of the OCN were calculated.

We also studied the gustatory, somatosensory, and integrative networks of COVID-19 patients and healthy controls. The gustatory network included bilateral insula, frontal operculum, postcentral gyrus, inferior OFC, pregenual anterior cingulate, medial orbitofrontal gyrus and thalamus.[18 19] The center coordinates of the gustatory seed regions (a sphere of 6mm radius) were at the (1) -36, 6 -2 (left insula) and (2) 38, 20, 2 (right insula).

Furthermore, the somatosensory and integrative networks were evaluated. The somatosensory network included primary and secondary somatosensory cortices and insula, while the integrative network comprised of the OFC, insula, inferior parietal lobule, middle and superior temporal gyrus.[12] The sizes of ROIs were 10mm radius spheres. The center coordinates of the seed ROIs in the somatosensory network were (1) -58, -42, 36 (left supramarginal) and (2) 58, -32, 34 (right supramarginal). For the integrative network, the center coordinates of the seed ROIs were (1) -34, 22, 10 (left insula) and (2) 39, 6, 2 (right insula). After the seed-based FC calculations, we overlapped the masks of the three networks with the FC maps. The threshold was set at 0.2 and maximum z value was standardized to 1.

Supplementary Note S3: Smell training via the electronic portable aromatic rehabilitation (EPAR) diffusers.

Four categories of essential oils [lemon (Citrus limon), eucalyptus (Eucalyptus radiata), geranium (Pelargonium graveolens), and cedarwood (Juniperus virginiana)] were used for smell training (ST), which were individually stored in four different EPAR diffuser units. Patients were instructed to conduct ST at a seated position, where the EPAR diffuser was placed at an arm's length with the stream of aerosolized essential oil directed upwards. During ST sessions, all four categories of essential oils were delivered via the EPAR diffusers for 20 seconds sequentially, providing a total of 80 seconds of olfactory stimulation three times per day.

Supplementary Table S1: Demographic and clinical characteristics of COVID-19 patients and controls.

	**COVID-19 patients (n=2)**		**Controls (n=2)**	
**Characteristics**	**Patient 1 (P1)**	**Patient 2 (P2)**	**Control 1 (C1)**	**Control 2 (C2)**
Age (years)	19	41	26	29
Gender	Male	Female	Male	Female
COVID-19 Disease Status	Mild	Mild	Not applicable	Not applicable
Respiratory condition				
SpO_2 (room air)	98%	97%		
Dyspnoea	No	No		
Cough	No	No		
Sputum production	No	No		
Radiological lung changes	Nil	Bilateral patchy ground glass opacities		
Systemic symptoms				
Fever	No	Yes		
Chills	No	No		
Rigor	No	No		
Malaise	No	No		
Myalgia	No	No		
Vomiting	No	No		
Diarrhoea	No	No		
SARS-CoV-2 Investigations				
RT-PCR (Ct value)	Positive (23.79)	Positive (22.06)	Negative	Negative
Date of RT-PCR virologic diagnosis	31 March 2020	16 March 2020		
Date of RT-PCR virologic clearance	2 May 2020	6 April 2020		
Serology (MN titre)	Positive (1:40)	Positive (1:40)	Negative (<1:10)	Negative (<1:10)
Nasoendoscopy Assessments	No anatomical abnormalities	No anatomical abnormalities	Not available	Not available
Olfactory Function Assessments				
Onset of Olfactory Dysfunction	25 March 2020	23 March 2020		
BTT score (baseline)				
Baseline	0.5	0.5	6.5	4.5
2 weeks	1.0	Not available	Not available	Not available
4 weeks	2.0	Not available	Not available	Not available
Smell Identification Test				

Baseline	Anosmia	Severe Microsmia	Not available	Not available
4 weeks	Severe Microsmia	Not available	Not available	Not available
Smoking Status	Non-smoker	Non-smoker	Non-smoker	Non-smoker
Drinking Status	Social drinker	Non-drinker	Non-drinker	Non-drinker
Past Surgical History				
Head injuries	Nil	Nil	Nil	Nil
Neurosurgery	Nil	Nil	Nil	Nil
Maxillofacial surgery	Nil	Nil	Nil	Nil
Past Medical History	GPH	GPH	GPH	GPH

BTT: butanol threshold test; COVID-19: coronavirus disease 2019; Ct value: cycle threshold value; GPH: good past health; MN titre: microneutralization assay titre; RT-PCR: reverse transcription polymerase chain reaction; SARS-CoV-2: severe acute respiratory syndrome coronavirus 2; SpO_2: oxygen saturation.

Supplementary Table S2: Sino-nasal outcome test (SNOT-22) of patient 1, before olfactory treatment.

(1) Considering how severe the problem is when you experience it and how often it happens, please rate each item below on how "bad" it is by circling the number that corresponds with how you feel using this scale: →	No Problem	Very Mild Problem	Mild or slight Problem	Moderate Problem	Severe Problem	Problem as bad as it can be	5 Most Important Items
1. Need to blow nose	0	×	2	3	4	5	
2. Nasal Blockage	×	1	2	3	4	5	
3. Sneezing	×	1	2	3	4	5	
4. Runny nose	0	×	2	3	4	5	
5. Cough	×	1	2	3	4	5	
6. Post-nasal discharge	×	1	2	3	4	5	
7. Thick nasal discharge	×	1	2	3	4	5	
8. Ear fullness	0	1	×	3	4	5	
9. Dizziness	×	1	2	3	4	5	
10. Ear pain	0	×	2	3	4	5	
11. Facial pain/pressure	0	×	2	3	4	5	
12. Decreased Sense of Smell/Taste	0	1	2	3	4	×	■
13. Difficulty falling asleep	×	1	2	3	4	5	
14. Wake up at night	×	1	2	3	4	5	
15. Lack of a good night's sleep	×	1	2	3	4	5	
16. Wake up tired	0	1	×	3	4	5	
17. Fatigue	×	1	2	3	4	5	
18. Reduced productivity	×	1	2	3	4	5	
19. Reduced concentration	×	1	2	3	4	5	
20. Frustrated/restless/irritable	×	1	2	3	4	5	
21. Sad	×	1	2	3	4	5	
22. Embarrassed	×	1	2	3	4	5	

(2) Please mark the most important items affecting your health (maximum of 5 items)

SNOT-20 Copyright © 1996 by Jay F. Piccirillo, M.D., Washington University School of Medicine, St. Louis, Missouri

SNOT-22 Developed from modification of SNOT-20 by National Comparative Audit of Surgery for Nasal Polyposis and Rhinosinusitis Royal College of Surgeons of England.

Supplementary Table S3: Sino-nasal outcome test (SNOT-22) of patient 1, after 4-weeks of olfactory treatment.

(1) Considering how severe the problem is when you experience it and how often it happens, please rate each item below on how "bad" it is by circling the number that corresponds with how you feel using this scale: →	No Problem	Very Mild Problem	Mild or slight Problem	Moderate Problem	Severe Problem	Problem as bad as it can be	5 Most Important Items
1. Need to blow nose	0	1	×	3	4	5	
2. Nasal Blockage	0	1	2	×	4	5	
3. Sneezing	0	×	2	3	4	5	
4. Runny nose	0	1	×	3	4	5	
5. Cough	×	1	2	3	4	5	
6. Post-nasal discharge	0	×	2	3	4	5	
7. Thick nasal discharge	0	1	×	3	4	5	
8. Ear fullness	0	1	2	×	4	5	■
9. Dizziness	×	1	2	3	4	5	
10. Ear pain	0	×	2	3	4	5	
11. Facial pain/pressure	×	1	2	3	4	5	
12. Decreased Sense of Smell/Taste	0	1	2	3	×	5	■
13. Difficulty falling asleep	0	×	2	3	4	5	■
14. Wake up at night	0	1	×	3	4	5	
15. Lack of a good night's sleep	0	×	2	3	4	5	
16. Wake up tired	0	1	×	3	4	5	■
17. Fatigue	0	×	2	3	4	5	
18. Reduced productivity	0	×	2	3	4	5	
19. Reduced concentration	×	1	2	3	4	5	
20. Frustrated/restless/irritable	×	1	2	3	4	5	
21. Sad	×	1	2	3	4	5	
22. Embarrassed	×	1	2	3	4	5	

(2) Please mark the most important items affecting your health (maximum of 5 items)

SNOT-20 Copyright © 1996 by Jay F. Piccirillo, M.D., Washington University School of Medicine, St. Louis, Missouri

SNOT-22 Developed from modification of SNOT-20 by National Comparative Audit of Surgery for Nasal Polyposis and Rhinosinusitis Royal College of Surgeons of England.

Supplementary Table S4: Sino-nasal outcome test (SNOT-22) of patient 2.

(1) Considering how severe the problem is when you experience it and how often it happens, please rate each item below on how "bad" it is by circling the number that corresponds with how you feel using this scale: →	No Problem	Very Mild Problem	Mild or slight Problem	Moderate Problem	Severe Problem	Problem as bad as it can be	5 Most Important Items
1. Need to blow nose	×	1	2	3	4	5	
2. Nasal Blockage	×	1	2	3	4	5	
3. Sneezing	×	1	2	3	4	5	
4. Runny nose	×	1	2	3	4	5	
5. Cough	×	1	2	3	4	5	
6. Post-nasal discharge	×	1	2	3	4	5	
7. Thick nasal discharge	×	1	2	3	4	5	
8. Ear fullness	×	1	2	3	4	5	
9. Dizziness	0	1	×	3	4	5	■
10. Ear pain	×	1	2	3	4	5	
11. Facial pain/pressure	×	1	2	3	4	5	
12. Decreased Sense of Smell/Taste	0	1	2	3	×	5	■
13. Difficulty falling asleep	0	×	2	3	4	5	
14. Wake up at night	×	1	2	3	4	5	
15. Lack of a good night's sleep	×	1	2	3	4	5	
16. Wake up tired	0	×	2	3	4	5	
17. Fatigue	0	1	×	3	4	5	
18. Reduced productivity	0	1	×	3	4	5	
19. Reduced concentration	0	×	2	3	4	5	
20. Frustrated/restless/irritable	0	1	2	3	×	5	■
21. Sad	0	1	2	3	×	5	■
22. Embarrassed	0	1	2	3	×	5	■

(2) Please mark the most important items affecting your health (maximum of 5 items)

SNOT-20 Copyright © 1996 by Jay F. Piccirillo, M.D., Washington University School of Medicine, St. Louis, Missouri

SNOT-22 Developed from modification of SNOT-20 by National Comparative Audit of Surgery for Nasal Polyposis and Rhinosinusitis Royal College of Surgeons of England.

Supplementary Table S5: Functional connectivity (FC) of the left and right caudate and the olfactory cortical network (OCN) regions.

	Left caudate seed			Right caudate seed		
**Subjects**	**Primary OCN regions**	**Secondary OCN regions**	**Combined OCN regions**	**Primary OCN regions**	**Secondary OCN regions**	**Combined OCN regions**
C–1	0.55	0.40	0.40	0.46	0.37	0.35
C–2	0.50	0.42	0.41	0.37	0.26	0.25
P1–1	0.35	0.22	0.19	0.28	0.11	0.09
P1–2	0.28	0.17	0.17	0.24	0.15	0.14
P1–3	0.26	0.23	0.21	0.26	0.14	0.15
P2–1	0.11	0.08	0.06	0.20	0.23	0.20

C–1: Control 1; C–2: Control 2; P1–1: Patient 1–baseline; P1–2: Patient 1–2 weeks of vitamin A and smell training; P1–3: Patient 1–additional 2 weeks of smell training, after 2 weeks of combination treatment with vitamin A and smell training; P2–1: Patient 2–baseline.

Supplementary Table S6: Volume of the olfactory bulb and tract.

	Volume of the olfactory bulb and tract (mm^3)	
**Subjects**	**Left side**	**Right side**
C–1	49.5	45
C–2	84	79.5
P1–1	37.5	22.5
P1–2	36	37.5
P1–3	42	49.5
P2–1	39	27

C–1: Control 1; C–2: Control 2; P1–1: Patient 1–baseline; P1–2: Patient 1–2 weeks of vitamin A and smell training; P1–3: Patient 1–additional 2 weeks of smell training, after 2 weeks of combination treatment with vitamin A and smell training; P2–1: Patient 2–baseline.

Supplementary Table S7: Cerebral blood flow (CBF) in the olfactory cortical network (OCN) regions.

	Cerebral blood flow (mL/100g/min)				
**Anatomical area**	**Healthy mean**	**P1–1**	**P1–2**	**P–3**	**P2–1**
**Primary OCN areas**					
Piriform cortex (left)	49.87	54.38	57.35	63.86	56.39
Piriform cortex (right)	53.18	53.13	59.04	58.97	54.69
Amygdala (left)	44.74	59.98	47.92	49.52	49.34
Amygdala (right)	40.47	46.01	45.12	38.83	49.99
**Secondary OCN areas**					
Orbitofrontal cortex (left, superior)	63.26	50.53	66.02	70.25	61.28
Orbitofrontal cortex (right, superior)	62.34	53.94	70.49	67.14	60.77
Orbitofrontal cortex (left, inferior)	55.94	51.10	60.49	66.21	57.65
Orbitofrontal cortex (right, inferior)	55.27	50.71	57.40	58.67	58.01
Orbitofrontal cortex (left, medial)	62.64	70.97	66.07	69.77	58.96
Orbitofrontal cortex (right, medial)	59.12	69.12	63.18	65.14	57.19
Gyrus rectus (left)	61.58	49.56	68.72	71.38	65.71
Gyrus rectus (right)	63.02	56.72	67.40	68.51	63.57
Insula (left)	52.99	55.65	53.30	58.20	54.73
Insula (right)	50.39	54.33	55.64	57.37	56.63
Anterior cingulate cortex (left)	54.73	58.90	64.33	66.02	61.20
Anterior cingulate cortex (right)	49.70	52.99	61.13	62.61	55.39
Hippocampus (left)	47.15	67.60	49.28	54.93	58.04
Hippocampus (right)	47.60	59.68	50.13	49.71	55.48
Parahippocampus (left)	50.23	53.69	52.72	62.24	57.06
Parahippocampus (right)	51.59	56.29	55.22	55.26	57.28
Caudate nucleus (left)	42.09	52.50	43.47	46.79	40.82
Caudate nucleus (right)	44.28	51.27	50.23	46.83	41.06
Putamen (left)	43.36	53.85	50.22	49.20	44.65
Putamen (right)	43.03	50.49	50.21	45.00	46.97
Temporal pole (left, superior)	53.38	53.34	46.97	56.03	47.49
Temporal pole (right, superior)	51.57	56.95	50.10	54.18	51.57
Temporal pole (left, middle)	55.09	48.98	56.79	61.64	54.66
Temporal pole (right, middle)	51.33	54.18	55.71	58.94	57.11

C–1: Control 1; C–2: Control 2; P1–1: Patient 1–baseline; P1–2: Patient 1–2 weeks of vitamin A and smell training; P1–3: Patient 1–additional 2 weeks of smell training, after 2 weeks of combination treatment with vitamin A and smell training; P2–1: Patient 2–baseline.

Supplementary Table S8: Butanol threshold test (BTT).

Step	Dilution step	1-butanol (mL)	Deionized water (mL)	1-butanol concentration (%)
1	1	20mL of 99.9% 1-butanol	480	4
2	2	50mL of dilution step 1	150	1
3	3	50mL of dilution step 2	150	0.25
4	4	50mL of dilution step 3	150	0.0625
5	5	50mL of dilution step 4	150	0.01563
6	6	50mL of dilution step 5	150	0.00391
7	7	50mL of dilution step 6	150	0.00098
8	8	50mL of dilution step 7	150	0.00024
9	9	50mL of dilution step 8	150	0.00006
10	10	50mL of dilution step 9	150	0.00002

10-steps serial dilution of 99.9% 1-butanol. Each BTT bottle contained 100mL of solutions, excess volume was discarded.

Supplementary Figures S1 | Magnetic resonance (MR) spectroscopy of the gyrus rectus and superior frontal cortex. (A) The position of the single voxel Point Resolved Spectroscopy (PRESS) was placed at the gyrus rectus and superior frontal cortex. (B) Healthy control. (C) Patient 2. Cho, choline; Cr, creatine; mI, myo-inositol; NAA, N-acetylaspartate.

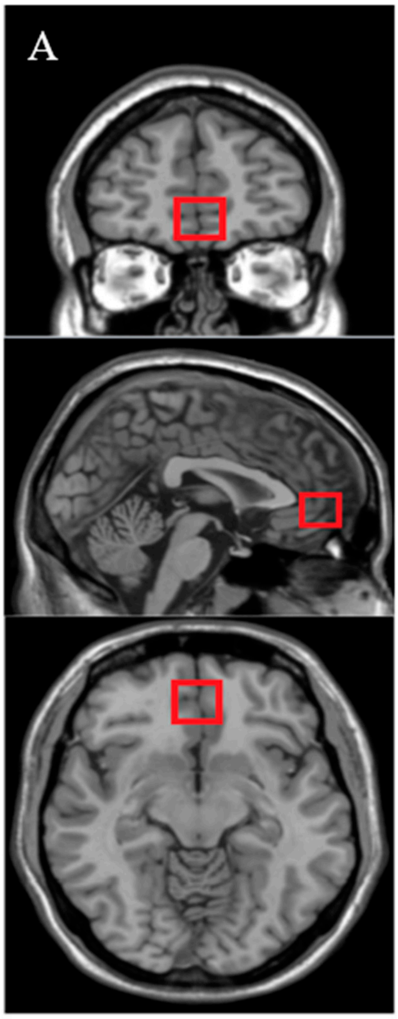
A

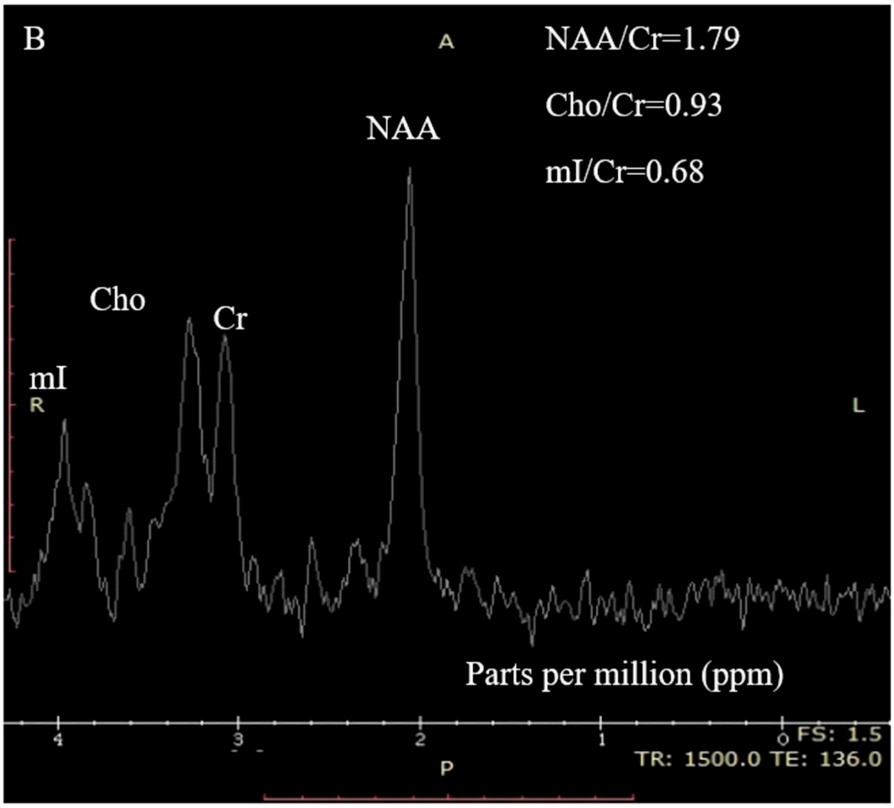
B
A
NAA/Cr=1.79
Cho/Cr=0.93
mI/Cr=0.68
NAA
Cho
Cr
mI
R
L
Parts per million (ppm)
4
3
2
1
0
FS: 1.5
TR: 1500.0 TE: 136.0
P

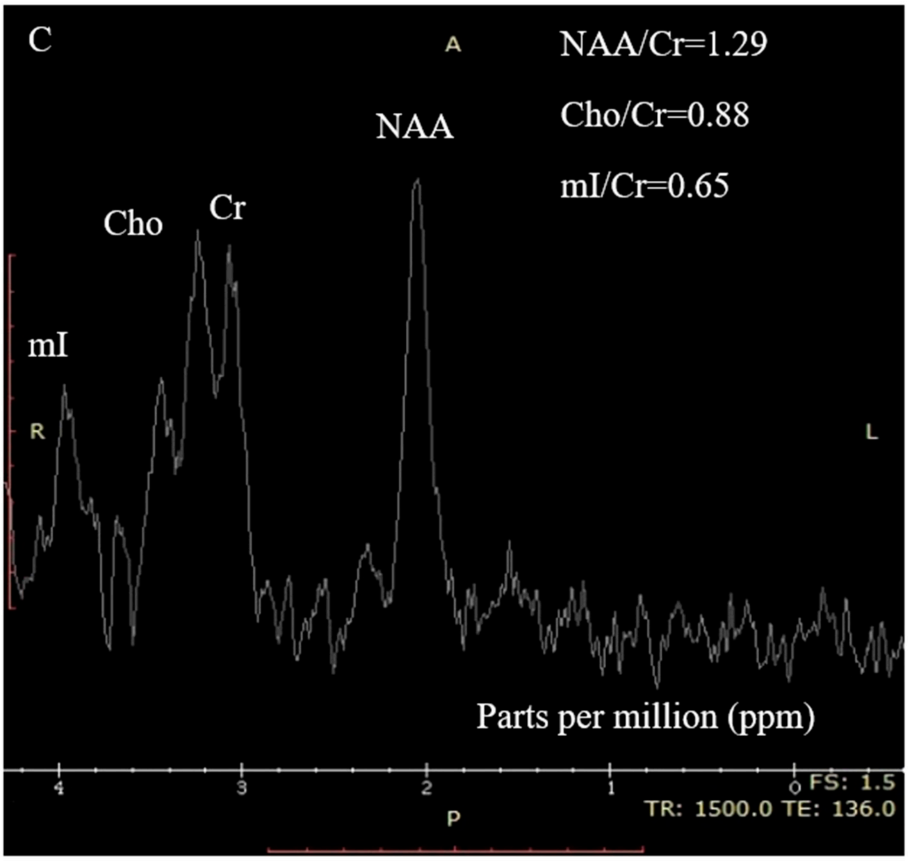

Supplementary Figures S2 | Electronic portable aromatic rehabilitation (EPAR) diffuser. (A) Correct usage, stream of aerosolised essential oil directed vertically upwards, away from the user's face. (B) Incorrect usage, stream of aerosolised essential oil directed at the user's face, which may cause transient irritation to the skin and mucosal surfaces.

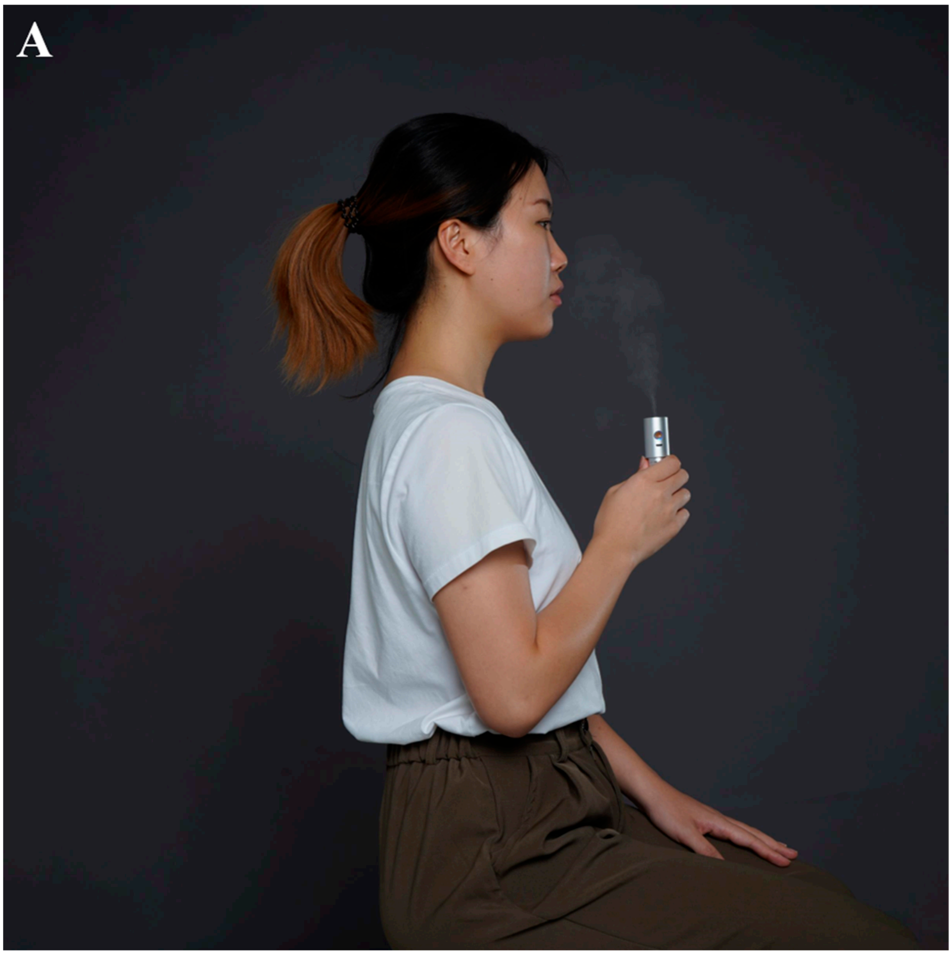
A

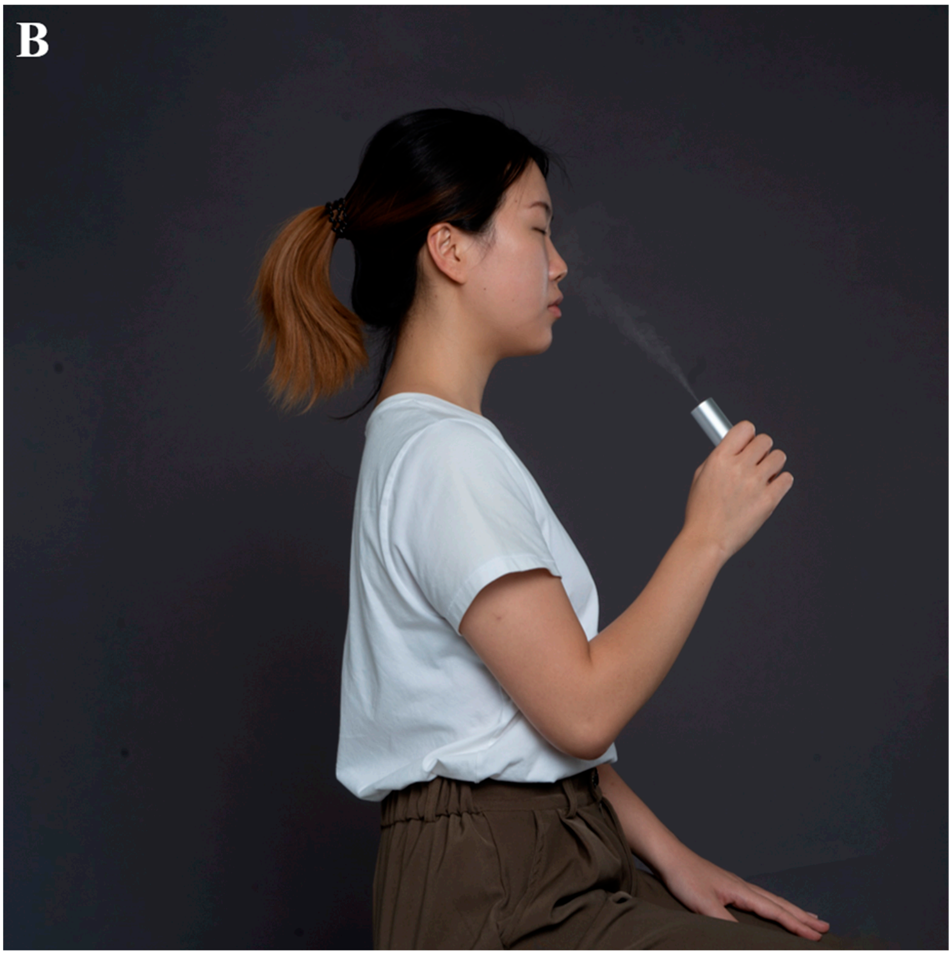
B

Supplementary Item S1: Olfactory function questionnaire.

**Basic patient characteristics**	
Level of education	
Occupation	
Smoking status	⊚ Non-smoker<br>⊚ Ex-smoker<br>⊚ Smoker<br><br>Detailed smoking history<br>• Age of first exposure to cigarette smoking: __________ (years)<br>• How many cigarettes smoked per day: ________ (pack per day)<br>• Any exposure to vaping or cigar smoking: yes ⊚ or no ⊚<br>• Any self-perceived alterations in the ability to smell during the period of smoking: **yes** ⊚ or **no** ⊚, comments: __________<br>• Age of smoking cessation (if applicable): __________ (years)<br>• Any self-perceived alterations in the ability to smell after cessation of smoking: **yes** ⊚ or **no** ⊚, comments: __________
Drinking status	⊚ Non-drinker<br>⊚ Occasional drinker<br>⊚ Social drinker<br><br>Detailed drinking history<br>• Frequency of alcohol consumption: ________ (glasses per week)<br>• Type of alcohol consumed: ______________________________________
Recreational drug use	⊚ No<br>⊚ Yes, comments: ______________________________ (cannabis, cocaine)
Chemical exposure	⊚ No<br>⊚ Yes, comments: ____
Past Medical history	⊚ No<br>⊚ Yes, comments: ___________________________ (nasal polyps, allergic rhinitis, diabetes mellitus, hypothyroidism, rheumatological conditions)
History of head trauma	⊚ No<br>⊚ Yes, comments: ____
History of neurosurgery or maxillofacial surgery	⊚ No<br>⊚ Yes, comments: ____
History of primary varicella zoster virus infection	⊚ No<br>⊚ Yes, comments: ___________________________ (age of onset), any history of zoster reactivation ____ (area affected).

**Smell History**	
Self-perceived smell impairments	◎ No<br>◎ Yes, date of onset: __________________ (dd / mm / year)
Anosmia	◎ No<br>◎ Yes
Hyposmia	◎ No<br>◎ Yes, comments: ____
Parosmia	◎ No<br>◎ Yes, comments: ____
Cacosmia	◎ No<br>◎ Yes, comments: ____

**Smell History (administered to COVID-19 patients with olfactory dysfunction)**	
Self-perceived improvements in smell function	◎ No<br>◎ Yes<br><br>Details of improvements<br>• Date of onset of improvements: __________________ (dd / mm / year)<br>• Comments: ____
Persistent smell impairments	◎ No<br>◎ Yes<br><br>Details of improvements<br>• Change in appetite: **yes** ◎ or **no** ◎<br>Comments: ____<br>• Change in mood: **yes** ◎ or **no** ◎<br>Comments: ____

Supplementary References:

1. Chung TW, Sridhar S, Zhang AJ, et al. Olfactory Dysfunction in Coronavirus Disease 2019 Patients: Observational Cohort Study and Systematic Review. *Open Forum Infect Dis* 2020;7(6):ofaa199. doi: 10.1093/ofid/ofaa199 [published Online First: 2020/06/18]

2. Malaty J, Malaty IA. Smell and taste disorders in primary care. *Am Fam Physician* 2013;88(12):852-9. [published Online First: 2013/12/25]

3. Piccirillo JF, Merritt MG, Jr., Richards ML. Psychometric and clinimetric validity of the 20-Item Sino-Nasal Outcome Test (SNOT-20). *Otolaryngol Head Neck Surg* 2002;126(1):41-7. doi: 10.1067/mhn.2002.121022 [published Online First: 2002/02/01]

4. Cain WS, Gent JF, Goodspeed RB, et al. Evaluation of olfactory dysfunction in the Connecticut Chemosensory Clinical Research Center. *Laryngoscope* 1988;98(1):83-8. doi: 10.1288/00005537-198801000-00017 [published Online First: 1988/01/01]

5. Doty RL, Shaman P, Dann M. Development of the University of Pennsylvania Smell Identification Test: a standardized microencapsulated test of olfactory function. *Physiol Behav* 1984;32(3):489-502. doi: 10.1016/0031-9384(84)90269-5 [published Online First: 1984/03/01]

6. To KK, Tsang OT, Leung WS, et al. Temporal profiles of viral load in posterior oropharyngeal saliva samples and serum antibody responses during infection by SARS-CoV-2: an observational cohort study. *Lancet Infect Dis* 2020;20(5):565-74. doi: 10.1016/s1473-3099(20)30196-1 [published Online First: 2020/03/28]

7. To KK-W, Cheng VC-C, Cai J-P, et al. Seroprevalence of SARS-CoV-2 in Hong Kong and in residents evacuated from Hubei province, China: a multicohort study. *The Lancet Microbe* 2020;1(3):e111-e18. doi: 10.1016/S2666-5247(20)30053-7

8. Yousem DM, Geckle RJ, Bilker WB, et al. Posttraumatic olfactory dysfunction: MR and clinical evaluation. *AJNR Am J Neuroradiol* 1996;17(6):1171-9. [published Online First: 1996/06/01]

9. Oz G, Alger JR, Barker PB, et al. Clinical proton MR spectroscopy in central nervous system disorders. *Radiology* 2014;270(3):658-79. doi: 10.1148/radiol.13130531 [published Online First: 2014/02/27]

10. Ashburner J. A fast diffeomorphic image registration algorithm. *Neuroimage* 2007;38(1):95-113. doi: 10.1016/j.neuroimage.2007.07.007 [published Online First: 2007/09/01]

11. Tzourio-Mazoyer N, Landeau B, Papathanassiou D, et al. Automated anatomical labeling of activations in SPM using a macroscopic anatomical parcellation of the MNI MRI single-subject brain. *Neuroimage* 2002;15(1):273-89. doi: 10.1006/nimg.2001.0978 [published Online First: 2002/01/05]

12. Kollndorfer K, Fischmeister FP, Kowalczyk K, et al. Olfactory training induces changes in regional functional connectivity in patients with long-term smell loss. *Neuroimage Clin* 2015;9:401-10. doi: 10.1016/j.nicl.2015.09.004 [published Online First: 2015/11/26]

13. Fjaeldstad A, Fernandes HM, Van Hartevelt TJ, et al. Brain fingerprints of olfaction: a novel structural method for assessing olfactory cortical networks in health and disease. *Sci Rep* 2017;7:42534. doi: 10.1038/srep42534 [published Online First: 2017/02/15]

14. Kollndorfer K, Kowalczyk K, Hoche E, et al. Recovery of olfactory function induces neuroplasticity effects in patients with smell loss. *Neural Plast* 2014;2014:140419. doi: 10.1155/2014/140419 [published Online First: 2014/12/30]
15. Karunanayaka P, Tobia MJ, Yang QX. Age-related resting-state functional connectivity in the olfactory and trigeminal networks. *Neuroreport* 2017;28(15):943-48. doi: 10.1097/wnr.0000000000000850 [published Online First: 2017/09/16]
16. Wang J, Eslinger PJ, Doty RL, et al. Olfactory deficit detected by fMRI in early Alzheimer's disease. *Brain Res* 2010;1357:184-94. doi: 10.1016/j.brainres.2010.08.018 [published Online First: 2010/08/17]
17. Bensafi M, Iannilli E, Gerber J, et al. Neural coding of stimulus concentration in the human olfactory and intranasal trigeminal systems. *Neuroscience* 2008;154(2):832-8. doi: 10.1016/j.neuroscience.2008.03.079 [published Online First: 2008/05/20]
18. Veldhuizen MG, Albrecht J, Zelano C, et al. Identification of human gustatory cortex by activation likelihood estimation. *Hum Brain Mapp* 2011;32(12):2256-66. doi: 10.1002/hbm.21188 [published Online First: 2011/02/10]
19. Chikazoe J, Lee DH, Kriegeskorte N, et al. Distinct representations of basic taste qualities in human gustatory cortex. *Nat Commun* 2019;10(1):1048. doi: 10.1038/s41467-019-08857-z [published Online First: 2019/03/07]
